# Supplementary material for: Screening to understand pregnancy preferences and offer referrals and treatment (SUPPORT): Results of a pilot quality improvement initiative
Source: PLoS One. 2024 Jul 29;19(7):e0303930. doi: 10.1371/journal.pone.0303930 (PMC11285907; doi:10.1371/journal.pone.0303930)
Supplement: Attachment — Submitted filename: Response to Reviewers.docx [file pone.0303930.s001.docx]

**Rebuttal Letter**

We thank both reviewers and the editorial team for their insightful feedback. Below, we describe the changes made in response to these comments. All text in black is cut-and-pasted from the review letter received. All text in red is our responses.

Editor comments:

Please ensure that figure 1 is attached to the manuscript

We have added this attachment as a separate .TIFF file labeled per guidelines.

Journal requirements:

When submitting your revision, we need you to address these additional requirements.

1. Please ensure that your manuscript meets PLOSONE's style requirements, including those for file naming. The PLOS ONE style templates can be found at

<https://journals>.plos.org/plosone/s/file?id=wjVg/PLOSOne_formatting_sample_main_body.pdf and

<https://journals.plos.org/plosone/s/file?id=ba62/PLOSOne_formatting_sample_title_authors_affiliations.pdf>

We have made several changes to conform with these guidelines, namely changing section headings to 18-point font, eliminating some use of italics, and moving Tables to the correct location.

3. Thank you for stating the following financial disclosure:

“This research was supported by internal funding provided through the Division of Women’s Health at Brigham and Women’s Hospital.”

Please state what role the funders took in the study.  If the funders had no role, please state: "The funders had no role in study design, data collection and analysis, decision to publish, or preparation of the manuscript."

If this statement is not correct you must amend it as needed.

Please include this amended Role of Funder statement in your cover letter; we will change the online submission form on your behalf.

We have added this statement to the Cover Letter.

5. Please include your tables as part of your main manuscript and remove the individual files. Please note that supplementary tables (should remain/ be uploaded) as separate "supporting information" files

We have added the tables to the manuscript file.

Reviewer #1: The manuscript reports on a pilot study to assess the feasibility of integrating pregnancy intention screening into routine care through electronic records prompts. The authors do a good job of explaining the context of the study and results. The study methods are appropriate for the research aim. I have a few suggestions to be considered.

The introduction goes into some detail about the importance of these questions for people with chronic diseases yet the rest of the paper including the title, abstract, aims and discussion do not mention this group being a focus of the study. Consider incorporating into the rest of the paper.

We have added the following content under “Setting”: While the primary care practices at Fish care for a wide variety of patients with and without chronic diseases, the co-location of many disease subspecialties in the clinic results in an overrepresentation of people with chronic conditions in the overall Fish patient population.

The setting of the study (that in a center for women’s health focusing on those with chronic conditions) could be better described in the abstract. Acronym AFAB is used in the abstract but not defined. The explanation that paused due to COVID is not really needed in abstract.

We have made the following changes to the abstract:

The Objective now reads: “To assess the feasibility of integrating a pregnancy intention assessment screening algorithm into the electronic medical record (EMR) at a multispecialty clinic focused on the health of women and people assigned female at birth (AFAB).”

We did not delete the mention of a pause due to COVID-19 because our follow-up period for analysis began after that pause. We want to accurately describe that we began the intervention in February 2020, but also be clear that our study data does not include the acute early COVID period, due to the 6-month pause.

Did clinicians prescreen if people were not able to become pregnant (other than those stated – documented hysterectomy or other sterilizing procedure) and may have been sensitive to questions about pregnancy?

No, there was no pre-screening of patients.

A patient demographics table or description is not included here but could help to understand the study population- including their age and race (as mentioned in limitations sections of discussion). It could also include % with a chronic condition if that information is available.

Thank you, we have added a new Table 1 with available demographic and visit characteristics.

If available include the number of clinicians involved in the study

We have added this information to the second paragraph under Results:

“During the intervention period, the SUPPORT screening algorithm was activated for 79 clinicians during visits for 4004 AFAB individuals ages 18-50.”

The paper would also benefit from some minor editorial changes

Line 180 Include the number of screenings in the text eg OB/GYN (353 screenings).
We have changed this sentence to read: “The greatest number of screenings were performed by OB/GYN (353 screenings) and primary care (258 screenings) clinicians.”

Line 197 – change (ref) to (reference group)

We have made this change.
• Line 189-192. Consider moving this sentence to start of next paragraph to keep the topic of contraception together.

We have made this change.

• Ensure using consistent terms in text and Tables. Primary care is used in text but Internal medicine used in Table 1.

We have changed several instances of “primary care” to “internal medicine” in the text. In the introduction and review of other studies, we generally retained “primary care” as a more expansive, and in that context more accurate, term.

• Since using aOR in the results text, use adjusted odds ratio in table 2 with a footnote that model is adjusted by linear age.

We have relabeled the column in this table “Adjusted Odds Ratio” and removed the rows for age, instead adding a footnote per this comment.

Reviewer #2: This study makes a helpful contribution to the literature on screening for reproductive desires in clinical practice in the United States. I have a few suggestions for clarity and accuracy within the text:

1) In the abstract, spell out the meaning of AFAB before using the acronym.

Thank you, we have made this change.

2) Line 66 says “women and pregnancy-capable individuals,” but I think all of the studies you cite (references 2-4) say they studied women or “adult females” without identifying pregnancy capability. I appreciate your effort to be gender inclusive, but I think in citing other studies it’s best to carry forward their language, and then you can make a separate note that it’s unknown whether people of all genders capable of pregnancy were included, or it’s unfortunate that they weren’t. (If you disagree and find that, for example, transgender men capable of pregnancy were included in these studies, then please ignore my comment.) In your sentence, “women with chronic diseases” would also be simpler/easier for the reader to follow.

We have changed this to say “female.” The reviewer is correct that the studies refer to females, not women, so we have aligned our language accordingly.

3) Line 74-75, for reader clarity, I think it would help to name/define the tools (PATH, OKQ) first and then briefly review the literature on them. As written, as each tool arises (e.g. PATH) the reader may not know what this is without prior introduction.

We have added this sentence to introduce the two screeners briefly before describing prior studies using them: “The proprietary One Key Question^TM^ (OKQ) screener queries patients regarding their desire to become pregnant in the next year. The more complex PATH questions (Pregnancy Attitudes, Timing and How important is pregnancy prevention) explore multiple dimensions of pregnancy attitudes, including the strength of motivation to avoid pregnancy.”

4) Line 111, “wide variety of clinical specializations and subspecialty clinics” sounds redundant

We have retained this phrase as written because “clinical specializations” refers to disciplines (such as gynecology) and “subspecialty clinics geared to specific populations” refers to special programs within or across disciplines (such as the PCOS program).

5) Line 139, “on site, at-the-elbow coaching” also sounds redundant

We deleted the phrase “at-the-elbow.”

6) Line 145 says one utility measurement is “likelihood of contraceptive documentation.” Can you define this more? Was this based on asking the patient? Or on med list, problem list, a checkbox, or other field? What contraceptive methods does it include (e.g. are withdrawal, fertility awareness, or abstinence included)?

Thank you for this excellent point. We have added these sentences:

“Contraceptive documentation was culled from medication lists and defined fields in the health or social history forms. Referrals were formal referrals in the EMR. We did not review free-text notes for either outcome.”

7) Line 194 says “using” contraception, but this seems different from the documentation outcome you’re measuring. (Unless the documentation is specifically of current contraceptive use and you feel this is a reliable indicator of patient behavior.)

The documentation is of current use of contraception as reported by the patient (and therefore documented by the clinical team). We do agree that it is important to remind the reader this is documented use, as opposed to actual use, which we cannot measure with this study design, so we changed this sentence to read: “individuals had higher odds of documented use of contraception on or within 90 days of the date of screening if they answered “I’m not sure”.”

8) Lines 218-220 comparing your findings to other studies: it would be helpful to write out in more detail what those other studies observed.

We have chosen not to add more details here as these are the same studies discussed, with a bit more detail, in the Introduction.

9) Line 247, in the Discussion you say your population lacked racial diversity, but you haven’t shared anything about the racial identities of the population.

Thank you for this important note. Per the other reviewer’s suggestion and in line with this comment, we have added demographic information on the patient population in a new Table 1.

While revising your submission, please upload your figure files to the Preflight Analysis and Conversion Engine (PACE) digital diagnostic tool, [https://pacev2.apexcovantage.com/](https://secure-web.cisco.com/1Ce2Hre9brhYlu8jeRNr01eJOmITjV-Lut3OB7aYjujlOlrFR7P3iWMdk0Rk6AEnmCHP_JxMdVKLGZ14OBIFXA5JObsg5UAW5RArUuOl2cdk5RXujTSL5uSYS1I6jEBuXJyTYoRLZ9Lm4hYWPxmBy-OdeGH_RZVdZAFUfk2UMko08-QrnHqyVsfb8CqonQwsEyRNMu35O5soHTc2sMgubZrlWVElDRTr_cmTEiT_Hx0TAEVHpXbfWPHusURaiVMvcpzGY-Uf6nXXwTOEUUj8Ci6dqfBQTsLjcQL_2HutKW9D04qUkzFWwynxgLEUjzyAY/https%3A%2F%2Fpacev2.apexcovantage.com%2F). PACE helps ensure that figures meet PLOS requirements. To use PACE, you must first register as a user. Registration is free. Then, login and navigate to the UPLOAD tab, where you will find detailed instructions on how to use the tool. If you encounter any issues or have any questions when using PACE, please email PLOS at [figures@plos.org](mailto:figures@plos.org). Please note that Supporting Information files do not need this step.

We have uploaded the Figure, accepted the proposed changes, downloaded the new file, and included that new file in our submission.
